# A PP2A-mediated feedback mechanism controls Ca^2+^-dependent NO synthesis under physiological oxygen

**DOI:** 10.1096/fj.201700211R

**Published:** 2017-07-31

**Authors:** Thomas P. Keeley, Richard C. M. Siow, Ron Jacob, Giovanni E. Mann

**Affiliations:** Cardiovascular Division, King’s British Heart Foundation Centre of Research Excellence, Faculty of Life Sciences and Medicine, King’s College London, London, United Kingdom

**Keywords:** endothelial cells, normoxia, hypoxia

## Abstract

Intracellular O_2_ is a key regulator of NO signaling, yet most *in vitro* studies are conducted in atmospheric O_2_ levels, hyperoxic with respect to the physiologic milieu. We investigated NO signaling in endothelial cells cultured in physiologic (5%) O_2_ and stimulated with histamine or shear stress. Culture of cells in 5% O_2_ (>5 d) decreased histamine- but not shear stress–stimulated endothelial (e)NOS activity. Unlike cells adapted to a hypoxic environment (1% O_2_), those cultured in 5% O_2_ still mobilized sufficient Ca^2+^ to activate AMPK. Enhanced expression and membrane targeting of PP2A-C was observed in 5% O_2_, resulting in greater interaction with eNOS in response to histamine. Moreover, increased dephosphorylation of eNOS in 5% O_2_ was Ca^2+^-sensitive and reversed by okadaic acid or PP2A-C siRNA. The present findings establish that Ca^2+^ mobilization stimulates both NO synthesis and PP2A-mediated eNOS dephosphorylation, thus constituting a novel negative feedback mechanism regulating eNOS activity not present in response to shear stress. This, coupled with enhanced NO bioavailability, underpins differences in NO signaling induced by inflammatory and physiologic stimuli that are apparent only in physiologic O_2_ levels. Furthermore, an explicit delineation between physiologic normoxia and genuine hypoxia is defined here, with implications for our understanding of pathophysiological hypoxia.—Keeley, T. P., Siow, R. C. M., Jacob, R., Mann, G. E. A PP2A-mediated feedback mechanism controls Ca^2+^-dependent NO synthesis under physiological oxygen.

The majority of *in vitro* experiments are routinely conducted in atmospheric O_2_ (∼20%), whereas cells *in vivo* experience significantly lower levels. Such artifactual hyperoxic conditions create a pro-oxidized environment that reduces cellular lifespan (1, 2), alters adaptive antioxidant defenses (3, 4), and may thus influence the translational relevance of *in vitro* findings. The blood-dissolved O_2_ level *in vivo* ranges from 13% in the pulmonary circulation to ∼3–5% in most microvascular capillary beds (5) and ∼3.7% in venous umbilical blood (6). Thus, HUVECs cultured in standard conditions *in vitro* are normally exposed to >4 times the O_2_ level experienced *in vivo*. We have reported that culturing HUVECs in 5% ambient O_2_ results in a cytosolic O_2_ level of ∼3.5% (4), more accurately replicating levels measured *in vivo* and with important consequences for cellular antioxidant defense pathways.

NO synthesis and metabolism are O_2_ dependent, a relationship critical in determining normal tissue O_2_ homoeostasis (7, 8). Thus, we investigated whether standard culture in hyperoxic conditions affects endothelial NO signaling. Upon exposure to hypoxic (∼1–2% O_2_) conditions, endothelial (e)NOS expression follows a biphasic pattern of an acute increase in mRNA and protein levels, peaking at ∼12 h (9), followed by a gradual decline as hypoxia persists beyond 24 h (10). These biphasic changes in mRNA and protein levels are paralleled by changes in NO production. To date, there are no reports on the long-term (>72 h) effects of physiologic O_2_ levels on NO signaling induced by inflammatory mediators or shear stress. In comparison to phosphorylation, limited data are available on the role of dephosphorylation in the regulation of eNOS. Whereas dephosphorylation at the inhibitory residues T495 (11) and S114 (12) by protein phosphatase (PP)-1 or -2B (calcineurin) has been described, acute dephosphorylation at stimulatory residues, such as S1177 and S633, is less well understood. PP2A has been shown to dephosphorylate eNOS at S1177 in response to long-term treatment with ceramide (13), endostatin (14), vasoinhibins (15), and proteasome inhibition (16). However, the mechanisms by which phosphatases regulate acute, transient Ca^2+^-stimulated eNOS phosphorylation at S1177 and S633 remain to be elucidated.

We report the first evidence that long-term culture of human endothelial cells in physiologic (5%) O_2_ levels induces a phenotype distinct from that observed in atmospheric conditions. By select comparison with paired cells cultured in 1% O_2_ (hypoxia), we further differentiate the cellular phenotype in normoxia (5% O_2_) from that often observed in genuine hypoxia. Specifically, we demonstrate that enhanced extranuclear PP2A activity in physiologic O_2_ (5%) provides more stringent regulation of eNOS activity in response to Ca^2+^-dependent inflammatory mediators. In contrast, the responses to fluid shear stress, which are not subject to acute serine-threonine phosphatase regulation, appear to be potentiated in physiologic O_2_ levels, emphasizing the importance of shear stress as the predominant physiological mediator of NO production. Our study further highlights the importance of physiologically relevant O_2_ levels for *in vitro* research to better correlate such findings with studies *in vivo*.

## MATERIALS AND METHODS

### Reagents and antibodies

Fura-2 AM was purchased from Teflabs (Austin, TX, USA) and Cal-520 AM from Stratech (Newmarket, United Kingdom). eNOS, PP2A-A (α/β), and PR72/130 antibodies were from Santa Cruz Biotechnologies (Dallas, TX, USA). Anti-PP2A-C and α-tubulin were from Millipore-Sigma (Watford, United Kingdom). Anti-phospho-eNOS S1177, T495, and S633; anti-phospho-AMPK T172; and total α-AMPK, anti-phospho-protein kinase B (Akt) S473 and total Akt, anti-phospho-ERK1/2 (T204/202) and total ERK1/2 antibodies were from Cell Signaling Technology (Danvers, MA, USA). Anti-HIF1-α antibody was from Novus Biologicals (Littleton, CO, USA). cGMP ELISA was from Cayman Chemical (Ann Arbor, MI, USA). The anti-SERCA-2 antibody (2D8) was kindly provided by Dr. Kalwant Authi (Cardiovascular Division, King’s College London). On-Target Plus PP2A-C (human) and control scrambled siRNA were from Dharmacon (Lafayette, CO, USA). All other chemicals were purchased from Millipore-Sigma.

### Culture of human primary endothelial cells

Umbilical cords from normal-term pregnancies were obtained from St. Thomas’ Hospital with informed participant consent and Research Ethics Committee approval (EC02/77, 15/EM/0290). HUVECs were isolated by collagenase digestion of cords from 73 participants to minimize potential variations in cell phenotype due to gender or ethnicity (17), and cultured in M199+20% fetal calf serum (FCS) (18). Human coronary artery endothelial cells (HCAECs) from a healthy 27-yr-old male donor (PromoCell, Heidelberg, Germany) were cultured in endothelial growth medium+5% FCS. Culture in physiologic (5%) or hypoxic (1%) O_2_ levels was achieved by transferring cells from a standard incubator (∼18% O_2_) into an O_2_-regulated workstation (Sci-tive; Baker-Ruskinn, Bridgend, United Kingdom) gassed to 5 or 1% O_2_. HUVECs were cultured in low O_2_ (10, 5, 3, or 1% O_2_) from passage 2 for a minimum of 5 d, to allow for cell proteome adaptation, and all subculture procedures performed in the appropriate percentage of O_2_. For siRNA transfection, cells were seeded at 35,000 cells per well and transfected for 48 h with scrambled or PP2A-C siRNA (50 nM). Cells at passage 3 were adapted to low serum (1% FCS) for 4 h before experimentation.

### Application of shear stress to cultured endothelial cells

Confluent HUVECs were acutely exposed to fluid shear stress, with a parallel-plate flow system (Ibidi, Martinsried, Germany). In brief, HUVECs were seeded onto μ-slides and allowed to adhere for 24 h. The μ-slides were then connected to a fluidic unit to generate a unidirectional laminar shear stress of 15 dyn/cm^2^.

### Intracellular Ca^2+^ measurements

Intracellular Ca^2+^ levels were measured in HUVECs loaded with Fura-2 AM (2 µM) and monitored in an O_2_ atmosphere-regulated plate reader (ClarioStar; BMG Labtech, Orteberg, Germany). Agonist or vehicle was injected with the integrated reagent injection system. Alternatively, for measurements of Ca^2+^ under shear stress, HUVECs were loaded with Cal-520 AM (2 µM) and fluorescence monitored at 480 excitation/520 emission on an inverted microscope (Lumascope; Etaluma, Carlsbad, CA, USA) within the Sci-tive workstation.

### Modeling of NO bioavailability

Mathematical modeling of NO bioavailability after addition of the NO donor (*Z*)-1-[*N*-(2-aminoethyl)-*N*-(2-ammonioethyl)amino]diazen-1-ium-1,2-diolate (DETA-NONOate) was performed using the method introduced in 1998 by Schmidt *et al.* (19), using previously published experimental parameters and accounting for the presence of cellular lipids (20) (see Supplemental Fig. S5 for details).

### Measurement of eNOS activity and intracellular cGMP production

eNOS enzymatic activity was determined by measuring the conversion of l-[^3^H]-arginine (4 µCi/ml) to l-[^3^H]-citrulline, separated in formic acid digests on Dowex 50W8 columns. eNOS activity was expressed as l-NAME inhibitable l-[^3^H]-citrulline production. cGMP production was assessed using a chemiluminescent ELISA (Arbor Assays, Ann Arbor, MI, USA) in the presence of the phosphodiesterase inhibitor IBMX (0.5 mM), with or without the eNOS inhibitor l-NAME (100 µM).

### Analysis of protein phosphorylation and expression by immunoblot analysis

HUVECs were grown to confluence and equilibrated to low serum (1% FCS) for 4 h. Cells were then equilibrated in Krebs buffer (mM: 131 NaCl_2_, 5.6 KCl, 20 HEPES, 25 NaHCO_3_, 5 d-glucose, 1 NaH_2_PO_4_, 0.1 l-arginine, 2 CaCl_2_, and 1 MgCl_2_) for a further 20 min before stimulation with histamine (10 µM), vehicle (double-distilled H_2_O), or laminar shear stress (15 dyn/cm^2^) for 2–30 min. Lysates were collected in SDS buffer containing phosphatase inhibitors. Equal amounts of protein (10–30 µg) were separated *via* SDS-PAGE. All immunoblot densities shown are relative to total protein (for phospho proteins) with β-actin used as an additional loading control.

### Immunofluorescent imaging of PP2A

HUVECs were washed twice in ice-cold PBS and fixed with 4% paraformaldehyde for 10 min. The cells were permeabilized in 0.1% Triton-X, blocked in 5% bovine serum albumin, and incubated with PP2A-A (1:100 dilution) for 1 h. A secondary antibody conjugated to FITC was added for an additional 1 h, and cells were counterstained with DAPI for 5 min. Samples were visualized on a Diaphot microscope (Nikon, Tokyo, Japan) coupled to an ORCA-03G camera (Hamamatsu, Shizuoka, Japan). Nuclear distribution of PP2A was determined by measuring PP2A staining intensity in a DAPI mask and expressed as a percentage of total intensity. On average, 5–6 regions of interest, containing 2–5 cells, were imaged per condition. In total, 107 cells from 4 different donors were analyzed.

### PP2A subcellular distribution and interactions with eNOS

Subcellular distribution of PP2A was assessed by immunofluorescence and coimmunoprecipitation (see below). PP2A–eNOS interactions were quantified by *in situ* proximity ligation (Duolink; Millipore-Sigma) (21). The cells were stained with PP2A and eNOS antibodies and incubated with PLA Plus and Minus probes, according to the manufacturer’s instructions. Nuclei were counterstained with DAPI and cells visualized with a ×40 objective. Six to 10 regions of interest were imaged per condition, with 1290 cells used for batch quantification with the analyze particles algorithm in ImageJ (National Institutes of Health, Bethesda, MD, USA). Results are expressed as a frequency distribution of PLA signals in individual cells and as mean PLA signals per cell for each condition and donor. Higher magnification (×60) images were used to visualize subcellular distribution of PLA signals. Coimmunoprecipitation of eNOS from 500 µg cell lysate was achieved by overnight incubation with protein A-labeled Dynabeads (Thermo Fisher Scientific, Loughborough, United Kingdom) conjugated to 1 µg eNOS antibody (Santa Cruz Biotechnology). SDS eluent was separated by PAGE and probed for PP2A-C content.

### Preparation of cytosolic and particulate subcellular fractions

HUVECs were washed twice in ice-cold PBS and scraped into Eppendorf tubes (Hamburg, Germany) in homogenization buffer (in mM: HEPES 10, sorbitol 340, EDTA 1, DTT 2, and protease and phosphatase inhibitor cocktails). Lysates were separated into cytosolic (Cav1/SERCA^−^) and particulate (Cav1/SERCA^+^, α-tubulin^−^) fractions by ultracentrifugation (22).

### Statistical analysis

Data are means ± sem of measurements in 3–11 different donors, with significance (*P* < 0.05) assessed with either a paired Student’s *t* test or 2-way ANOVA with Bonferroni *post hoc* analysis. Where appropriate, results were analyzed by regression analysis. Unless stated otherwise, the effects of both histamine and shear stress were significant (*P* < 0.05) *vs.* vehicle.

## RESULTS

### Maintenance of endothelial cells in 5% O_2_ does not stabilize HIF1α

**Figure 1.**
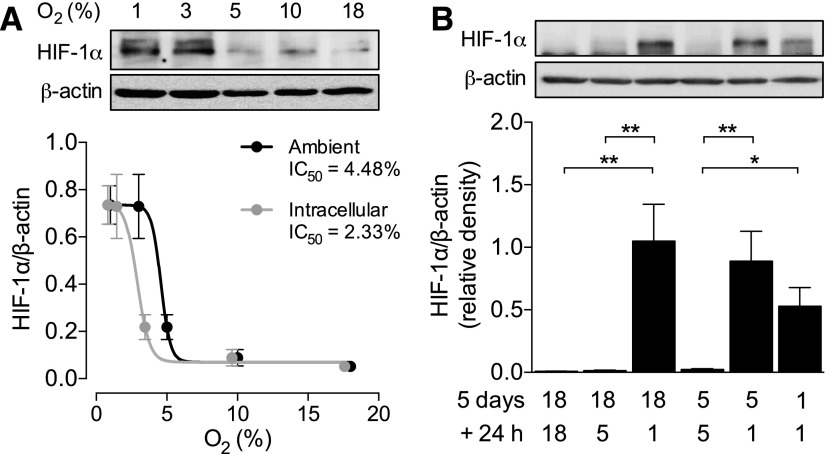
HIF-1α stabilization in HUVECs at various oxygen levels. *A*) HUVECs were cultured in 18, 10, 5, 3, or 1% O_2_ for at least 5 d, and lysates were immunoblotted for expression of HIF-1α. Means ± sem (*n* = 4) were plotted against either the ambient or intracellular O_2_ level [as determined previously (4)], and a sigmoidal curve was fitted for determination of the IC_50_. *B*) HUVECs cultured in 18, 5, or 1% O_2_ for 5 d were then cultured for a further 24 h in either the same or lower O_2_ levels, and expression of HIF-1α was determined as before. Representative immunoblots are provided above and densitometric analysis of HIF-1α expression relative to the loading control β-actin below. Data are means ± sem (*n* = 4–5 different donors). *P < 0.05, **P < 0.01.

Culture facilitates the formation of a significant O_2_ gradient between atmospheric and intracellular O_2_ levels minimized *in vivo* because of the flow of blood. We have previously demonstrated that culture in 5% ambient O_2_ is necessary to compensate for this artifactual gradient *in vitro*, thus replicating intracellular O_2_ levels observed in HUVECs *in vivo* (∼3.7%) (4). We first sought to understand how culture in these conditions relates to traditionally hypoxic environments, using the stabilization of HIF-1α as a readout. When HUVECs were cultured in a range of different O_2_ levels (18, 10 5, 3, and 1% O_2_) for more than 5 d, HIF-1α stabilization was evident only when the ambient O_2_ level was ≤3–4% (**Fig. 1*A***), with minimal expression evident at 5% O_2_ even when exposed acutely (Fig. 1*B*). When replotted against the corresponding mean intracellular O_2_ level [as determined previously (4)], there was a leftward shift in the sigmoidal curve with a reduction in IC_50_ from 4.5 to 2.3% O_2_. This shift is of particular physiologic significance, because blood oxygen (Po_2_) level of 4.5% is normoxic for most cell types *in vivo*, yet 2.3% O_2_ encroaches on hypoxia for endothelial cells. Hence intracellular, and not the ambient O_2_ level, is the important parameter when designing an *in vitro* model of physiologic normoxia. In the context of the current work, these data confirm that culture in 5% O_2_ does not induce a traditionally hypoxic phenotype in HUVECs.

### Altered Ca^2+^ mobilization after culture in 5% O_2_

**Figure 2.**
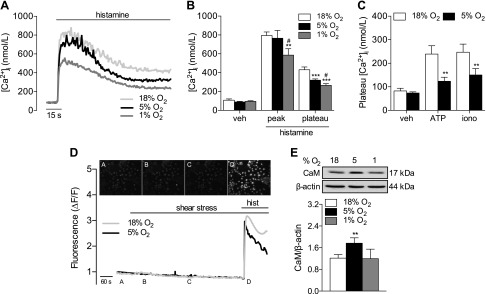
Attenuated Ca^2+^ mobilization in human endothelial cells cultured in various levels of O_2_. HUVECs were maintained at 18% O_2_ or cultured in 5 or 1% O_2_ for at least 5 d. Cells were stimulated with histamine (10 µM), and [Ca^2+^]_i_ was monitored. *A*) Ca^2+^ transients in HUVECs stimulated with histamine (10 µM) in 18, 5, or 1% O_2_. *B*) Average peak and plateau [Ca^2+^]_i_ values in response to histamine. *C*) Plateau [Ca^2+^]_i_ levels in response to ATP (10 µM) or ionomycin (0.1 µM). *D*) HUVECs were loaded with Cal520-AM and then exposed to 15 dyn/cm^2^ unidirectional shear stress for 10 min, followed by an acute stimulation with histamine. Representative images are provided from HUVECs at 18% O_2_. *E*) Calmodulin (CaM) expression in HUVECs in 18, 5, or 1% O_2_. Representative immunoblots and analysis of CaM expression relative to β-actin. Treatment with histamine was significant (*P* < 0.05 *vs.* respective control) in relevant panels. Data are means ± sem (*n* = 4–11 different donors). **P < 0.01, ***P < 0.001 *vs.* 18% O_2_; ^#^*P* < 0.05 *vs.* 5% O_2_.

Acute NO production evoked by inflammatory mediators is mediated by an initial increase in intracellular Ca^2+^, and thus we measured mobilization of Ca^2+^ after stimulation of HUVECs with histamine (10 µM). Histamine induced a typical biphasic change in intracellular Ca^2+^ [Ca^2+^]_i_ that was characterized by a sharp increase followed by a sustained plateau (**Fig. 2*A***). Peak [Ca^2+^]_i_ responses were similar in HUVECs cultured in 18 or 5% O_2_, but significantly lower in cells in 1% O_2_ (Fig. 2*B*), whereas the plateau phase was markedly reduced at both 5 and 1% O_2_. This effect was not due to differences in histamine- or inositol triphosphate–related signaling, as expression of the H1 histamine receptor was unaltered after long-term culture in 5% O_2_ (data not shown), and comparable results were obtained in HUVECs challenged with ATP (10 µM) or ionomycin (0.1 µM; Fig. 2*C*). Similar results were also observed in HCAECs (Supplemental Fig. S1*A*). Laminar shear stress (15 dyn/cm^2^) did not elicit an increase in mean intracellular Ca^2+^ over a 10 min period in HUVECs in either 18 or 5% O_2_ (Fig. 2*D*). As most endothelial cells experience only ≤1% O_2_ during periods of low- to no-flow ischemia, cells maintained at 1% O_2_ were not exposed to shear stress. We also observed significantly higher expression of calmodulin (CaM) in HUVECs cultured in 5% O_2_ compared with cells cultured in at either 18 or 1% O_2_ (Fig. 2*E*).

### Activation of kinases in endothelial cells in physiologic O_2_ levels

**Figure 3.**
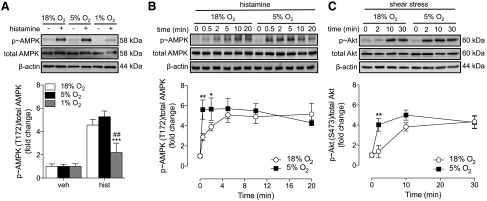
Activation of kinases in HUVECs cultured in various O_2_ levels. HUVECs were maintained at 18% O_2_ or cultured in 5 or 1% O_2_ for at least 5 d. Cells were then stimulated with histamine (10 µM) for 2 min (*A*) or 0–20 min (*B*), or laminar shear stress (15 dyn/cm^2^) for 0–30 min (*C*). Lysates were immunoblotted for p-AMPK or p-Akt and expressed relative to total kinase levels and β-actin as loading controls. Data are means ± sem (*n* = 5–6 different donors). Treatment with histamine or shear stress was significant (*P* < 0.05 *vs.* respective control) in relevant panels. *P < 0.05, **P < 0.01, ***P < 0.001 *vs.* 18% O_2_; ^##^*P* < 0.01 *vs.* 5% O_2_.

Because AMPK acts downstream of the H1 receptor in a Ca^2+^-dependent manner (23), we investigated whether alterations in Ca^2+^ mobilization were paralleled by activation of AMPK. Culturing HUVECs in 5% O_2_ had no significant effect on basal kinase expression or phosphorylation (Supplemental Fig. S2*A*). When cells were cultured in 1% O_2_, no significant phosphorylation of AMPK (**Fig. 3*A***) or ERK1/2 (data not shown) was detected in response to histamine. In contrast, in both 18 and 5% O_2_ histamine (0–20 min) caused rapid AMPK phosphorylation, reaching maximum levels after 5 min (Fig. 3*B*). HUVECs cultured in 5% O_2_ responded more rapidly to histamine, with significantly higher AMPK phosphorylation detected after 0.5 and 2 min. Although histamine also induced phosphorylation of ERK1/2, no differences were detected between cells cultured in 18 or 5% O_2_ (Supplemental Fig. S2*B*). Akt is largely responsible for mediating eNOS activation at basal Ca^2+^ levels, especially in response to physiologic stimuli, such as shear stress (24). In this study, shear stress (0–30 min) elicited a marked and sustained phosphorylation of Akt in both 18 and 5% O_2_, and HUVECs cultured in 5% O_2_ responded more rapidly with significantly higher Akt phosphorylation detected at 2 min (Fig. 3*C*).

### eNOS phosphorylation in physiologic O_2_ levels

**Figure 4.**
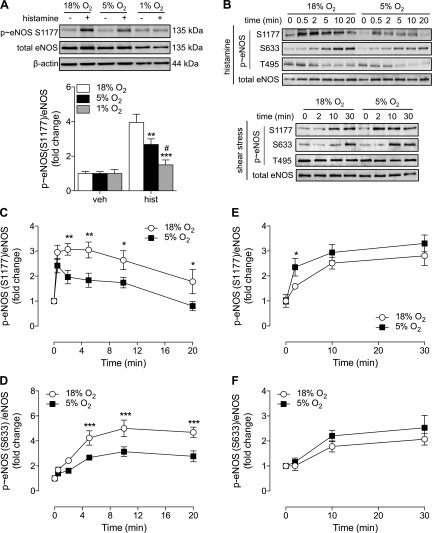
eNOS phosphorylation in HUVECs cultured in various O_2_ levels. HUVECs were maintained at 18% O_2_ or cultured in 5 or 1% O_2_ for at least 5 d. Cells were then stimulated with histamine (10 µM) for 2 min (*A*) or 0.5–20 min (*B*–*D*), or shear stress for 0–30 min (*B*, *E*, *F*), and phosphorylation of eNOS at S1177 (*A–C*, *E*) and S633 (*B*, *D*, *F*) (human sequence) assessed by immunoblot analysis relative to total eNOS, a β-actin loading control, or both. Data are expressed as fold change and are means ± sem (*n* = 4–6 different donors). Treatment with histamine or shear stress was significant (*P* < 0.05 *vs.* respective control) in relevant panels, except at 1% O_2_ in *A*. *P < 0.05, **P < 0.01; ***P < 0.001 *vs.* 18% O_2_; ^#^*P* < 0.05 *vs.* 5% O_2_.

Differential Ca^2+^ mobilization did not appear to affect the extent of AMPK or ERK1/2 activation in HUVECs cultured in 5% O_2_, and thus we examined whether eNOS phosphorylation would be similarly unaffected. eNOS protein expression (Supplemental Fig. S2*C*) and basal phosphorylation at 2 key stimulatory residues, S1177 and S633, (Supplemental Fig. S2*A*), were unaltered during culture in 5% O_2_. Treatment with histamine (2 min) led to rapid eNOS-S1177 phosphorylation in HUVECs at both 18 and 5% O_2_, but not at 1% O_2_ (**Fig. 4*A***). Notably, phosphorylation of eNOS-S1177 in response to histamine was similar in cells in 18 or 5% O_2_ after 30 s but then declined more rapidly over 2–15 min in cells cultured in 5% O_2_ (Fig. 4*C*, and at 2 min in HCAECs; Supplemental Fig. S1*B*). S633 was also phosphorylated in response to histamine, again significantly less in cells in 5% O_2_ (Fig. 4*D*). Dephosphorylation of eNOS at the inhibitory site T495 is known to enhance enzyme activity (11), but no difference in histamine-stimulated T495 dephosphorylation was observed in HUVECs at either 18 or 5% O_2_ (data not shown). Exposing HUVECs to shear stress (0–30 min) resulted in a time-dependent increase in eNOS-S1177 phosphorylation, with significantly higher phosphorylation detected after 2 min in cells cultured in 5% O_2_ (Fig. 4*E*). As with S1177, shear stress also led to rapid phosphorylation of S633, which was negligibly affected by the ambient O_2_ level (Fig. 4*F*). Furthermore, shear stress did not affect eNOS-T495 phosphorylation (data not shown).

### Consequences for endothelial NO and cGMP production

**Figure 5.**
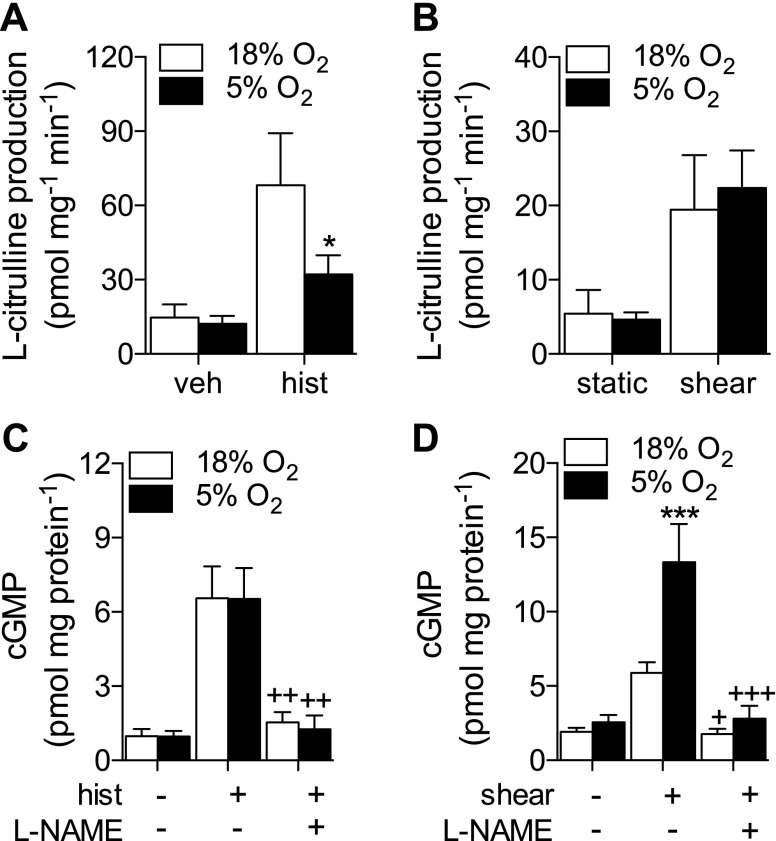
NO and cGMP production in HUVECs cultured in 5% O_2_. HUVECs were maintained at 18% O_2_ or cultured in 5% O_2_ for at least 5 d. Cells were pretreated in the absence or presence of the eNOS inhibitor l-NAME (100 µM) for 30 min before stimulation with histamine or shear stress. *A*, *B*) eNOS activity after stimulation with histamine (10 µM, 5 min) (*A*) or shear stress (15 dyn/cm^2^, 10 min) (*B*), plotted as l-NAME inhibitable l-[^3^H]citrulline production. *C*, *D*) cGMP production in response to histamine (10 µM, 5 min) (*C*) or shear stress (15 dyn/cm^2^, 10 min) (*D*). Data are means ± sem (*n* = 5–9 different donors). Treatment with histamine or shear stress was significant (*P* < 0.05 *vs.* respective control) in relevant panels. *P < 0.05, ***P < 0.001 *vs.* 18% O_2_; ^+^*P* < 0.05, ^++^*P* < 0.01, ^+++^*P* < 0.001 *vs.* histamine/shear stress alone.

To evaluate whether alterations in eNOS phosphorylation translated to enzymatic activity and thus NO production, histamine- and shear stress–stimulated eNOS activity and cGMP production were assessed in HUVECs cultured in 5% O_2_. Histamine stimulated an increase in eNOS activity that was significantly attenuated in HUVECs at 5% O_2_ (**Fig. 5*A***). In contrast, the increase in eNOS activity upon shear stress stimulation was similar at the 2 O_2_ levels (Fig. 5*B*). l-NAME inhibitable cGMP generation increased significantly after stimulation with either histamine or shear stress (Fig. 5*C*, *D*, respectively). Although histamine-stimulated eNOS activity was lower in 5% O_2_, cGMP production remained similar. In comparison, shear stress–stimulated cGMP production was significantly higher in cells at 5% O_2_ (Fig. 5*D*), despite similar levels of eNOS activity. It is well established that chronic (>24 h) hypoxia reduces eNOS activity and subsequent cGMP production in endothelial cells (9, 25). In agreement with this, we observed negligible increases in eNOS activity and significantly lower cGMP generation in response to histamine at 1% O_2_ (Supplemental Fig. S3*A*, *B*). Furthermore, expression of soluble guanylate cyclase was unchanged after long-term culture in 5 or 1% O_2_ (Supplemental Fig. S3*C*).

**Figure 6.**
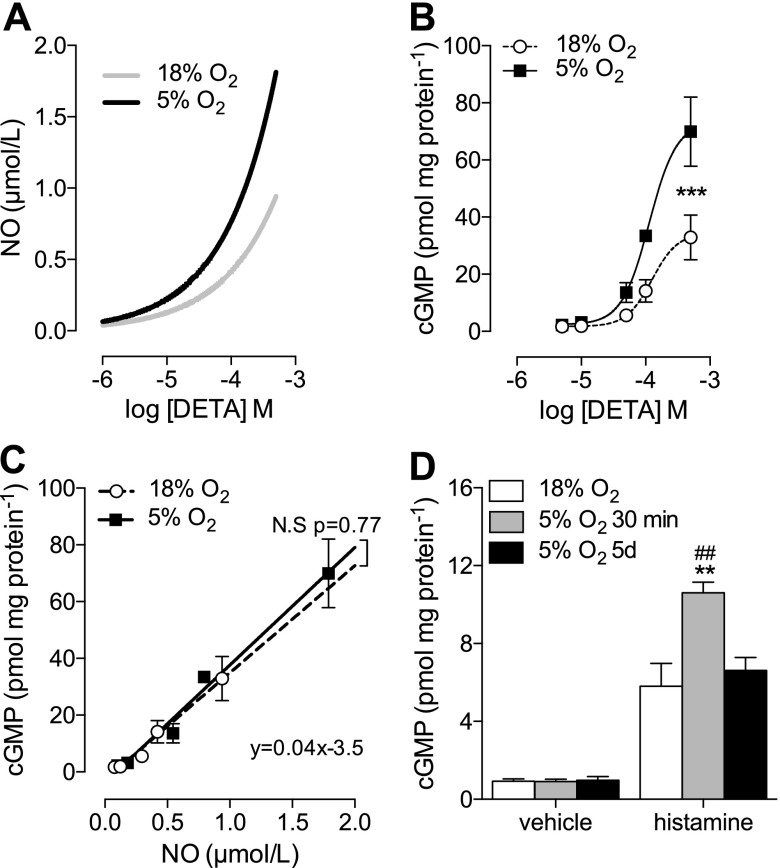
Impact of O_2_ on NO bioavailability in endothelial cells. *A*) Modeling of NO release from different levels of DETA after 10 min of decomposition. *B*) HUVECs were maintained at 18% O_2_ or cultured in 5% O_2_ for at least 5 d. Cells were treated with DETA (0–500 µM, 10 min), and cGMP production was assayed. Vehicle (NaOH 0.05%) had no effect on cGMP production (not shown). *C*) Correlation of predicted [NO] with measured cGMP levels. Differences in neither slope nor intercept were significant when 18 and 5% O_2_ were fitted separately, so one equation represents both O_2_ levels. *D*) Histamine (10 µM, 5 min) stimulated cGMP levels in HUVECs cultured in 18% O_2_ and then exposed acutely to 5% O_2_ for 30 min. Treatment with histamine was significant (*P* < 0.05 *vs.* respective control). Data are means ± sem (*n* = 3–9 different donors). **P < 0.01, ***P < 0.001 *vs.* 18% O_2_; ^##^*P* < 0.01 *vs.* 5% O_2_ (5 d).

O_2_ levels have been shown to be a key determinant of NO bioavailability (7, 8, 20), given that NO radicals react rapidly with molecular O_2_ to produce NO_2_^−^. A mathematical model of NO generation by DETA and removal by reaction with O_2_ (19) shows that the resulting NO concentration is higher in 5% O_2_ at any given concentration of DETA (**Fig. 6*A***). In addition, cGMP production in HUVECs after a 10-min stimulation with DETA (0–500 µM) was significantly higher in cells at 5% O_2_ (Fig. 6*B*). When cGMP production was plotted against calculated [NO] over a range of DETA concentrations (Fig. 6*C*), there was a strong linear relationship that did not vary significantly between O_2_ levels. To frame this in a physiological context, we took HUVECs cultured in 18% O_2_ and exposed them to 5% O_2_ for only 20 min, long enough for intracellular O_2_ to re-equilibrate (4) without evoking changes in eNOS activity. We hypothesized that an increase in NO bioavailability at 5% O_2_ levels without a change in eNOS activity would result in increased cGMP production, which was indeed observed (Fig. 6*D*).

### PP2A is responsible for enhanced eNOS dephosphorylation in physiologic O_2_

**Figure 7.**
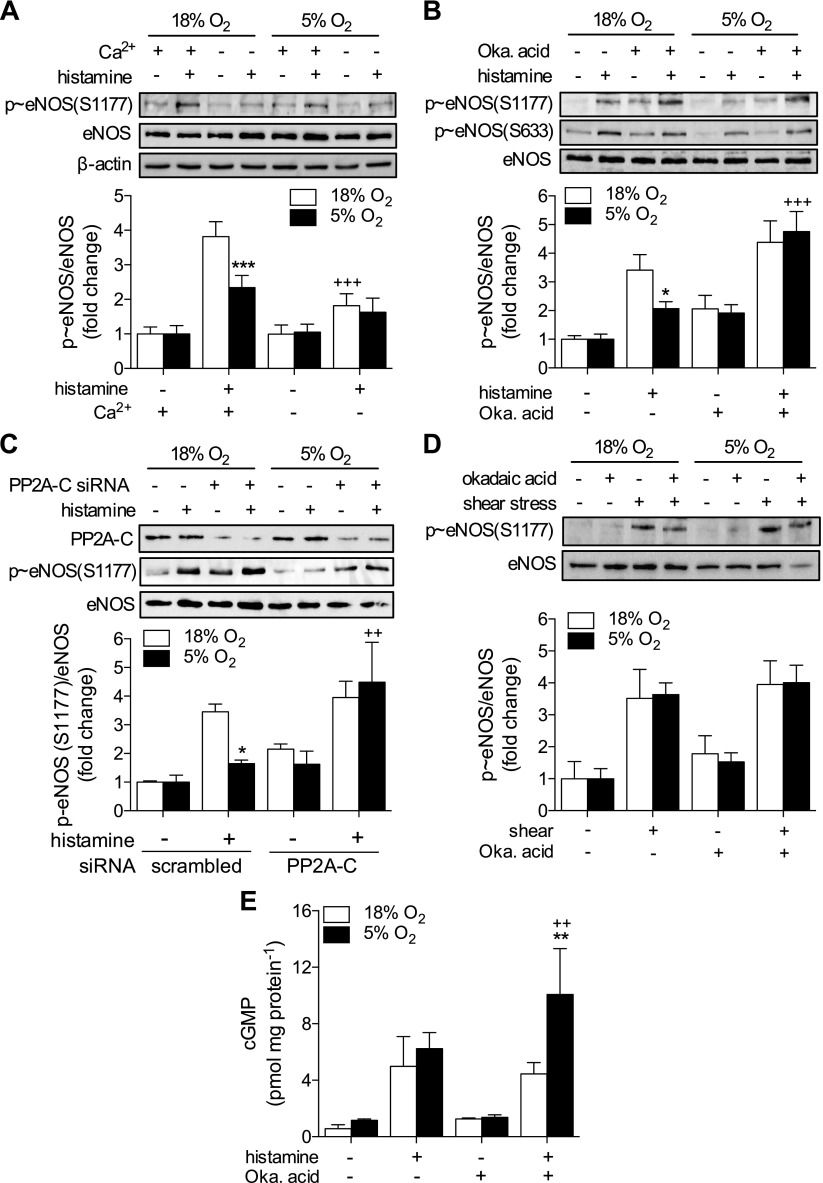
PP2A is responsible for histamine-stimulated eNOS dephosphorylation. HUVECs were maintained at 18% O_2_ or cultured in 5% O_2_ for at least 5 d. *A*) Cells were stimulated with histamine (10 µM, 2 min), with or without external Ca^2+^, and eNOS-S1177 phosphorylation was assessed. *B*) HUVECs were pretreated with the PP2A inhibitor okadaic acid (100 nM, 30 min), and phosphorylation of eNOS-S1177/S633 was assessed in response to histamine (10 µM, 5 min). *C*) HUVECs were transfected with scrambled (−) or PP2A-C siRNA and then stimulated with histamine. *D*) eNOS phosphorylation at S1177 in HUVECs pretreated with okadaic acid and then subjected to shear stress (15 dyn/cm^2^, 10 min). *E*) cGMP production in response to histamine (10 µM, 5 min) after pretreatment with okadaic acid. Representative immunoblots and densitometric analyses for p-eNOS expression relative to total eNOS and a β-actin loading control (data not shown in all panels). Data are expressed as fold change and are means ± sem (*n* = 4–6 different donors). Treatment with histamine or shear stress was significant (*P* < 0.05 *vs.* respective control) in relevant panels. *P < 0.05, **P < 0.01, ***P < 0.001 *vs.* 18% O_2_; ^+^*P* < 0.05, ^++^*P* < 0.01, ^+++^*P* < 0.001 *vs.* histamine alone/+Ca^2+^.

The discrepancy between kinase activation (Fig. 3) and eNOS phosphorylation (Fig. 4) in response to histamine in 5% O_2_ suggests a role for increased dephosphorylation. Most dephosphorylation of serine/threonine is catalyzed by PP1, -2A, or -2B (26). Although all 3 have been demonstrated to target eNOS, only PP2A has been shown to dephosphorylate S1177, whereas residues T495 and S114 are strongly targeted by PP2B and possibly PP1 (11, 27). The lack of upstream kinase activation at 1% O_2_ in response to histamine (Fig. 3*A*) likely explains the absence of eNOS phosphorylation (Fig. 4*A*), and therefore the role of phosphatases was investigated only in cells cultured in 18 and 5% O_2_. The effect of culture at 5% O_2_ was Ca^2+^-dependent, because in nominally Ca^2+^-free conditions, the dependence of S1177 phosphorylation on O_2_ was no longer apparent (**Fig. 7*A***), indicating the involvement of either PP2A or -2B. To investigate this further, HUVECs, pretreated with the PP2A inhibitor okadaic acid (100 nM, 30 min), were stimulated with histamine for a further 5 min. This concentration is the lowest possible dose that demonstrates phosphatase inhibition in cells, thereby limiting nonspecific effects on other phosphatases (PP1, -4, and -6) (28). Treatment with okadaic acid increased basal S1177 phosphorylation and rescued histamine-stimulated S1177 phosphorylation in cells in 5% O_2_ (Fig. 7*B*). Similar effects were observed for eNOS-S633 phosphorylation and after siRNA knockdown of PP2A–C (Fig. 7*C*). In contrast, pretreatment with the PP2B inhibitor FK506 had no effect on eNOS S1177/S633 phosphorylation (data not shown). Pretreatment with okadaic acid had no significant effect on shear stress–stimulated S1177 phosphorylation (Fig. 7*D*). cGMP production in HUVECs treated with histamine+okadaic acid was significantly higher in 5% O_2_ compared to 18% O_2_ (Fig. 7*E*).

### PP2A expression and subcellular location in endothelial cells in physiologic O_2_ levels

**Figure 8.**
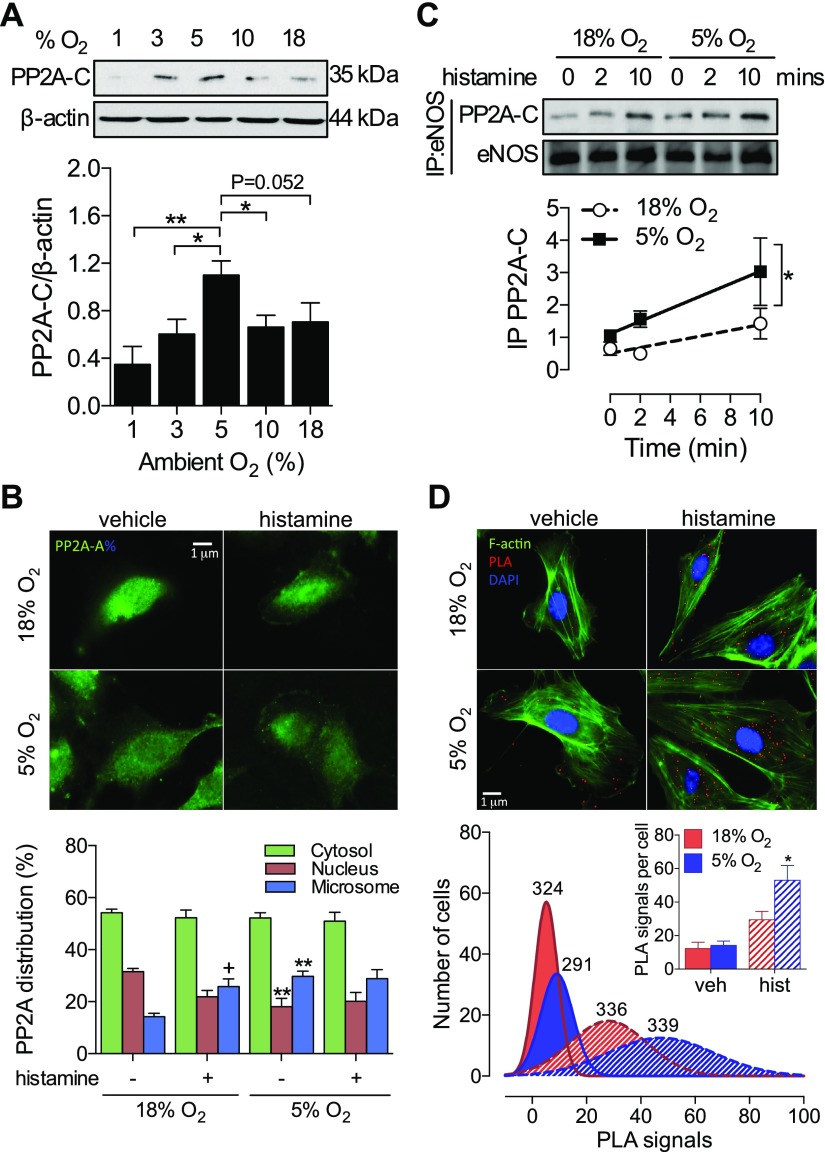
PP2A is responsible for histamine-stimulated eNOS dephosphorylation. HUVECs were maintained at 18% O_2_ or cultured in 10, 5, 3, or 1% O_2_ for at least 5 d. *A*) Representative immunoblot and densitometric analysis of PP2A-C expression relative to β-actin. *B*) Analysis of PP2A subcellular distribution in response to histamine stimulation (10 µM, 5 min) using immunofluorescence and ultracentrifugation (Supplemental Fig. S4). DAPI costaining not shown for clarity. *C*, *D*) Colocalization of eNOS and PP2A-C assessed by coimmunoprecipitation (*C*) and proximity ligation assay (*D*). Red dots indicate physical protein interaction. The total number of cells is indicated above each frequency distribution; data are means ± sem. PLA signals per cell shown in the inset. All other data represent means ± sem from 3 to 9 different donors. *P < 0.05, **P < 0.01 *vs.* 18% O_2_; ^+^*P* < 0.05 *vs.* vehicle.

A scaffold (A) and catalytic (C) subunit form the basal PP2A heterodimer, with substrate specificity and activity conferred by the choice of regulatory (B) subunit association (26). Notably, the expression of PP2A-C exhibited a bell-shaped distribution across a range of ambient O_2_ levels (1–18% O_2_), peaking at 5% and falling steeply as intracellular O_2_ levels became hypoxic (**Fig. 8*A***). The expression of PP2A-A and a Ca^2+^-sensitive B subunit (PR72) were unchanged, as was PP2B-C (data not shown). Previous studies have revealed translocation of PP2A to the plasma membrane, where it dephosphorylates eNOS (13, 16). Thus, we investigated whether PP2A-mediated eNOS dephosphorylation in cells cultured in 5% O_2_ could be explained by increased membrane localization. Because the PP2A-C subunit was induced by culture in 5% O_2_, we examined the localization of its dimer-forming partner PP2A-A (Fig. 8*B*). eNOS was found almost exclusively within the microsomal fraction (Supplemental Fig. S4), reflecting its known distribution near the plasma membrane or Golgi apparatus (29). In 18% O_2_, PP2A was predominantly (∼50%) found within the cytosolic fraction, with a smaller percentage associated with the nucleus, consistent with its role in cell cycle progression (30, 31). Moreover, histamine induced a redistribution from the nucleus to the microsome (Fig. 8*B*). In cells cultured in 5% O_2_, a similar redistribution was already apparent in unstimulated cells, and histamine did not induce a further redistribution. This basal redistribution in 5% O_2_ resulted in a more rapid interaction with eNOS, as reflected by an increased coimmunoprecipitation of PP2A-C with eNOS (Fig. 8*C*) and direct interaction assessed *via* *in situ* proximity ligation (21) (Fig. 8*D*).

## DISCUSSION

We have presented compelling evidence that *in vitro* data obtained at 5% O_2_ reveal functionally different NO signaling in endothelial cells from that observed in atmospheric O_2_. Long-term (>5 d) culture in physiologic normoxia highlights a novel negative-feedback mechanism regulating Ca^2+^-dependent eNOS activity, in which PP2A rapidly targets eNOS after Ca^2+^ mobilization to initiate dephosphorylation. Such feedback was not apparent during exposure to shear stress, providing clear delineation between physiologic and inflammatory pathways of NO production in endothelial cells. Genuine hypoxia (1% O_2_), in contrast to physiologic normoxia (5% O_2_), diminished responses to histamine at every stage of eNOS signaling from initial Ca^2+^ mobilization to cGMP production, consistent with previous reports (9, 10, 32–35).

The concept of physiologic normoxic cell culture has received increasing attention in recent years, with reports in primary lymphocytes (3, 36), neurons (37, 38), and stem cells (39–41) directly addressing the artifactual conditions in which cells are routinely cultured. In such studies, it is often demonstrated that cells exhibit less spontaneous or stimulated apoptosis (38), have less phenotypic differentiation during prolonged culture *ex vivo* (3), and have greater viability and regenerative capacity when transplanted into damaged organs *in vivo* (39, 40). Our previous findings in human endothelial cells (4) and in the present study illustrate a significant functional impact of culture at physiologic O_2_, with important implications for understanding endothelial cell responses to endogenous and pharmacological agents, as well as biomechanical forces. A picture is beginning to emerge in which long-term readaptation to physiologically relevant O_2_ levels *in vitro* dramatically alters cellular phenotype, with significant impact for translation of findings to animal models and the clinic.

In the present study, histamine and shear stress were used as classic endothelial cell stimuli to investigate Ca^2+^-sensitive and -insensitive pathways of NO generation in different O_2_ levels. Although a transient minor increase in [Ca^2+^]_i_ after flow initiation is sometimes observed in endothelial cells (42, 43), the Ca^2+^-insensitive nature of eNOS activation by this stimulus is defined by the lack of effect of removing external Ca^2+^ (44–46). The transient nature of histamine-stimulated eNOS activation contrasts with the gradual yet sustained activation observed during exposure to shear stress, indicating divergent signaling pathways. Because kinase activation in response to histamine was sustained (Fig. 3), the transience of eNOS phosphorylation implies a delayed dephosphorylation in cells cultured in 18% O_2_.

The finding that the culturing cells in 5% O_2_ significantly lowers plateau [Ca^2+^]_i_, whereas leaving peak responses largely unaffected is similar to findings reported by Østergaard *et al.* (10). A reduced plateau [Ca^2+^]_i_ was also observed in response to ATP or ionomycin treatment, indicating that the effect of culture in 5% O_2_ is not confined to H1-receptor activation and instead lies at a point of convergence downstream of inositol metabolism and receptor-mediated Ca^2+^ mobilization. CaM expression was found to be significantly increased after culture in 5% O_2_ (Fig. 2*E*), in line with previously published evidence (47). Cytosolic CaM concentrations are a key rate-limiting step in Ca^2+^-mediated signal transduction (48) and eNOS activation (49), and hence increased CaM expression may partially compensate for reductions in Ca^2+^ mobilization. Similarly, a lack of such a compensatory increase in CaM expression in 1% O_2_ may in part explain why hypoxic cells show little activation upon histamine stimulation. Increases in CaM may also provide an explanation for the enhanced AMPK phosphorylation in HUVECs at 5% O_2_, with Ca^2+^/CaM-dependent kinase II known to be an AMPK kinase (50).

Our experiments in human endothelial cells during physiologic normoxia have revealed a novel Ca^2+^-sensitive negative-feedback mechanism in regulating eNOS activation orchestrated by PP2A. In response to histamine, but not to shear stress, PP2A is rapidly recruited to eNOS and mediates the dephosphorylation at S1177 and, as demonstrated in this study for the first time, also at S633. This finding provides a mechanistic explanation of why Ca^2+^-dependent stimuli result in transient eNOS phosphorylation. In cells cultured in 1% O_2_, no upregulation of PP2A-C (Fig. 8*A*) or histamine-stimulated dephosphorylation was observed (data not shown), so this mechanism cannot explain the hypoxic reduction in eNOS phosphorylation, which is most likely due to severe global cellular dysfunction. Several members of the PP2A-B subunit family have been identified as Ca^2+^-sensitive, including the CaM-associated striatin (51) and the B family (PR70/72/130), which contain 2 EF-hand motifs and therefore can directly bind free Ca^2+^ (52). As B subunits regulate PP2A substrate specificity (53), the recruitment of one of these subunits likely mediates the relatively selective targeting of eNOS over other proposed PP2A substrates. Furthermore, members of the PP2A-B subunit family have been observed within the microsomal domain (54), providing an explanation for the similar distribution of active PP2A:eNOS complexes observed in the present study (Fig. 8). The enhanced basal microsomal distribution of PP2A in conjunction with increased expression of the catalytic subunit observed at 5% O_2_ may facilitate more rapid interaction with eNOS, which itself migrates toward the Golgi after stimulation (29).

To our knowledge, we report the first evidence of a relationship between cytosolic O_2_ levels and PP2A-C expression. Typically, PP2A subunit expression is regulated by translational autoregulation (55), although the absence of a change in basal phosphatase activity observed in our study suggests a different regulatory mechanism. It is unlikely that the PPPC2A mRNA promoter sequence contains a consensus hypoxia–response element (predicted based on sequence analysis; UCSC Genome Browser, University of California, Santa Cruz, CA, USA), and our observation that the expression of PP2A-C and HIF-1α show generally opposing responses to ambient O_2_ (Fig. 1*A* *vs.* Fig. 8*A*) suggest that PP2A-C expression is not regulated by traditional hypoxia signaling pathways. Rather, it seems likely that upregulation of PP2A-C specifically in physiologic normoxia (5% O_2_) may be a reflection of the cellular redox state, with both hyperoxia (18% O_2_) and hypoxia (1% O_2_) associated with higher levels of oxidative stress (56, 57). Further work is warranted to better understand the relationship between cellular redox state and PP2AC protein expression.

Using a combination of mathematical modeling and cell-based *in vitro* experiments, we highlight an inverse relationship between [O_2_] and [NO], first reported by Lancaster and colleagues (7, 20). In the present study, this relationship is framed within a dynamic physiologic context, examining its role in modulating the vascular response to stimulation. Accordingly, endothelial cells cultured in 5% O_2_ still generated an equivalent cGMP in response to histamine, despite reduced eNOS activity, whereas equivalent eNOS activity in response to shear stress generated considerably higher cGMP levels in 5% O_2_. Endothelial cells cultured in 1% O_2_ exhibited significant dysfunction upstream of soluble guanylate cyclase (Figs. 2, 3, and 4) and therefore did not capitalize fully on the reduced O_2_ conditions. Although other factors also contribute to the half-life of NO, such as the oxidation state of cytochrome *c* oxidase (58) and reactive oxygen species generation (59), these generally have a higher contribution in hyperoxic conditions (18% O_2_) and would therefore exaggerate only the existing predictions.

The data presented here highlight a clear disparity between the cellular responses to normoxia and hypoxia that extend beyond the specific focus of the current work. In this context, the following important points may be concluded based on our findings: *1*) when examined under the auspice of adaptation (>5 d) and not acute exposure (<24 h), the cellular response to reduced O_2_ levels is not, as previously thought, proportional to the magnitude of reduction in oxygen levels; *2*) changes in the expression of a protein during genuine hypoxia (1% O_2_) may be more appropriately determined when compared to physiologic normoxia rather than room air, as we demonstrated for CaM (Fig. 2*E*) and PP2A-C (Fig. 8*A*) expression; and *3*) effects of reducing cytosolic O_2_ levels within the physiologic range can still be readily observed in the absence of significant HIF-1α stabilization (Fig. 1).

In summary, our findings highlight several physiologic readouts significantly altered when cells are cultured in physiologic O_2_. Specific to this study, culture in 5% O_2_ facilitated the identification a novel negative feedback mechanism regulating Ca^2+^-sensitive NO production. Given its central role, changes in the physiology of PP2A in 5% O_2_, from subunit expression to subcellular distribution, have widespread implications for cell physiology. Advances in our understanding of physiology must be accompanied by adaptations in the way we study it, and thus we propose considering physiologic O_2_ levels as a significant advancement in routine cell culture.

## Supplementary Material

Supplemental Data

## Abbreviations

Akt: protein kinase B
[Ca^2+^]_i_: intracellular Ca^2+^
CaM: calmodulin
DETA NONOate: (*Z*)-1-[*N*-(2-aminoethyl)-*N*-(2-ammonioethyl)amino]diazen-1-ium-1,2-diolate
eNOS: endothelial NOS
FCS: fetal calf serum
HCAEC: human coronary artery endothelial cell
HIF: hypoxia-inducible factor
IBMX: 3-isobutyl-1-methylxanthine
NAME: *N*^G^-nitro-l-arginine methyl ester
PLA: proximity ligation assay
PP: protein phosphatase

## References

[1] Balin A. K., Goodman D. B., Rasmussen H., Cristofalo V. J. (1977) The effect of oxygen and vitamin E on the lifespan of human diploid cells in vitro. J. Cell Biol. 74, 58–67 874002 10.1083/jcb.74.1.58 PMC2109860

[2] Parrinello S., Samper E., Krtolica A., Goldstein J., Melov S., Campisi J. (2003) Oxygen sensitivity severely limits the replicative lifespan of murine fibroblasts. Nat. Cell Biol. 5, 741–747 12855956 10.1038/ncb1024 PMC4940195

[3] Atkuri K. R., Herzenberg L. A., Niemi A.-K., Cowan T., Herzenberg L. A. (2007) Importance of culturing primary lymphocytes at physiological oxygen levels. Proc. Natl. Acad. Sci. USA 104, 4547–4552 17360561 10.1073/pnas.0611732104 PMC1838638

[4] Chapple S. J., Keeley T. P., Mastronicola D., Arno M., Vizcay-Barrena G., Fleck R., Siow R. C. M., Mann G. E. (2016) Bach1 differentially regulates distinct Nrf2-dependent genes in human venous and coronary artery endothelial cells adapted to physiological oxygen levels. Free Radic. Biol. Med. 92, 152–162 26698668 10.1016/j.freeradbiomed.2015.12.013

[5] Tsai A. G., Johnson P. C., Intaglietta M. (2003) Oxygen gradients in the microcirculation. Physiol. Rev. 83, 933–963 12843412 10.1152/physrev.00034.2002

[6] Riley R. J., Johnson J. W. (1993) Collecting and analyzing cord blood gases. Clin. Obstet. Gynecol. 36, 13–23 7679616 10.1097/00003081-199303000-00005

[7] Thomas D. D., Liu X., Kantrow S. P., Lancaster J. R. Jr (2001) The biological lifetime of nitric oxide: implications for the perivascular dynamics of NO and O2. Proc. Natl. Acad. Sci. USA 98, 355–360 11134509 10.1073/pnas.011379598 PMC14594

[8] Victor V. M., Nuñez C., D’Ocón P., Taylor C. T., Esplugues J. V., Moncada S. (2009) Regulation of oxygen distribution in tissues by endothelial nitric oxide. Circ. Res. 104, 1178–1183 19407240 10.1161/CIRCRESAHA.109.197228

[9] Takemoto M., Sun J., Hiroki J., Shimokawa H., Liao J. K. (2002) Rho-kinase mediates hypoxia-induced downregulation of endothelial nitric oxide synthase. Circulation 106, 57–62 12093770 10.1161/01.cir.0000020682.73694.ab

[10] Østergaard L., Stankevicius E., Andersen M. R., Eskildsen-Helmond Y., Ledet T., Mulvany M. J., Simonsen U. (2007) Diminished NO release in chronic hypoxic human endothelial cells. Am. J. Physiol. Heart Circ. Physiol. 293, H2894–H2903 17720765 10.1152/ajpheart.01230.2006

[11] Fleming I., Fisslthaler B., Dimmeler S., Kemp B. E., Busse R. (2001) Phosphorylation of Thr(495) regulates Ca(2+)/calmodulin-dependent endothelial nitric oxide synthase activity. Circ. Res. 88, E68–E75 11397791 10.1161/hh1101.092677

[12] Kou R., Greif D., Michel T. (2002) Dephosphorylation of endothelial nitric-oxide synthase by vascular endothelial growth factor: implications for the vascular responses to cyclosporin A. J. Biol. Chem. 277, 29669–29673 12050171 10.1074/jbc.M204519200

[13] Zhang Q.-J., Holland W. L., Wilson L., Tanner J. M., Kearns D., Cahoon J. M., Pettey D., Losee J., Duncan B., Gale D., Kowalski C. A., Deeter N., Nichols A., Deesing M., Arrant C., Ruan T., Boehme C., McCamey D. R., Rou J., Ambal K., Narra K. K., Summers S. A., Abel E. D., Symons J. D. (2012) Ceramide mediates vascular dysfunction in diet-induced obesity by PP2A-mediated dephosphorylation of the eNOS-Akt complex. Diabetes 61, 1848–1859 22586587 10.2337/db11-1399 PMC3379648

[14] Urbich C., Reissner A., Chavakis E., Dernbach E., Haendeler J., Fleming I., Zeiher A. M., Kaszkin M., Dimmeler S. (2002) Dephosphorylation of endothelial nitric oxide synthase contributes to the anti-angiogenic effects of endostatin. FASEB J. 16, 706–708 11978735 10.1096/fj.01-0637fje

[15] García C., Aranda J., Arnold E., Thébault S., Macotela Y., López-Casillas F., Mendoza V., Quiroz-Mercado H., Hernández-Montiel H. L., Lin S.-H., de la Escalera G. M., Clapp C. (2008) Vasoinhibins prevent retinal vasopermeability associated with diabetic retinopathy in rats via protein phosphatase 2A-dependent eNOS inactivation. J. Clin. Invest. 118, 2291–2300 18497878 10.1172/JCI34508 PMC2391065

[16] Wei Q., Xia Y. (2006) Proteasome inhibition down-regulates endothelial nitric-oxide synthase phosphorylation and function. J. Biol. Chem. 281, 21652–21659 16737962 10.1074/jbc.M602105200

[17] Lorenz M., Koschate J., Kaufmann K., Kreye C., Mertens M., Kuebler W. M., Baumann G., Gossing G., Marki A., Zakrzewicz A., Miéville C., Benn A., Horbelt D., Wratil P. R., Stangl K., Stangl V. (2015) Does cellular sex matter? Dimorphic transcriptional differences between female and male endothelial cells. Atherosclerosis 240, 61–72 25756910 10.1016/j.atherosclerosis.2015.02.018

[18] Rowlands D. J., Chapple S., Siow R. C. M., Mann G. E. (2011) Equol-stimulated mitochondrial reactive oxygen species activate endothelial nitric oxide synthase and redox signaling in endothelial cells: roles for F-actin and GPR30. Hypertension 57, 833–840 21300668 10.1161/HYPERTENSIONAHA.110.162198 PMC3086276

[19] Schmidt K., Desch W., Klatt P., Kukovetz W. R., Mayer B. (1998) Release of NO from donor compounds: a mathematical model for calculation of NO concentrations in the presence of oxygen. Methods Mol. Biol. 100, 281–289 10907014 10.1385/1-59259-749-1:281

[20] Liu X., Miller M. J., Joshi M. S., Thomas D. D., Lancaster J. R. Jr (1998) Accelerated reaction of nitric oxide with O2 within the hydrophobic interior of biological membranes. Proc. Natl. Acad. Sci. USA 95, 2175–2179 9482858 10.1073/pnas.95.5.2175 PMC19287

[21] Söderberg O., Gullberg M., Jarvius M., Ridderstråle K., Leuchowius K.-J., Jarvius J., Wester K., Hydbring P., Bahram F., Larsson L.-G., Landegren U. (2006) Direct observation of individual endogenous protein complexes in situ by proximity ligation. Nat. Methods 3, 995–1000 17072308 10.1038/nmeth947

[22] Bokkala S., el-Daher S. S., Kakkar V. V., Wuytack F., Authi K. S. (1995) Localization and identification of Ca2+ATPases in highly purified human platelet plasma and intracellular membranes. Evidence that the monoclonal antibody PL/IM 430 recognizes the SERCA 3 Ca2+ATPase in human platelets. Biochem. J. 306, 837–842 7702581 10.1042/bj3060837 PMC1136596

[23] Thors B., Halldórsson H., Clarke G. D., Thorgeirsson G. (2003) Inhibition of Akt phosphorylation by thrombin, histamine and lysophosphatidylcholine in endothelial cells: differential role of protein kinase C. Atherosclerosis 168, 245–253 12801607 10.1016/s0021-9150(03)00127-8

[24] Dimmeler S., Fleming I., Fisslthaler B., Hermann C., Busse R., Zeiher A. M. (1999) Activation of nitric oxide synthase in endothelial cells by Akt-dependent phosphorylation. Nature 399, 601–605 10376603 10.1038/21224

[25] Kourembanas S., McQuillan L. P., Leung G. K., Faller D. V. (1993) Nitric oxide regulates the expression of vasoconstrictors and growth factors by vascular endothelium under both normoxia and hypoxia. J. Clin. Invest. 92, 99–104 8326022 10.1172/JCI116604 PMC293541

[26] Shi Y. (2009) Serine/threonine phosphatases: mechanism through structure. Cell 139, 468–484 19879837 10.1016/j.cell.2009.10.006

[27] Harris M. B., Ju H., Venema V. J., Liang H., Zou R., Michell B. J., Chen Z. P., Kemp B. E., Venema R. C. (2001) Reciprocal phosphorylation and regulation of endothelial nitric-oxide synthase in response to bradykinin stimulation. J. Biol. Chem. 276, 16587–16591 11340086 10.1074/jbc.M100229200

[28] Favre B., Turowski P., Hemmings B. A. (1997) Differential inhibition and posttranslational modification of protein phosphatase 1 and 2A in MCF7 cells treated with calyculin-A, okadaic acid, and tautomycin. J. Biol. Chem. 272, 13856–13863 9153244 10.1074/jbc.272.21.13856

[29] Fulton D., Fontana J., Sowa G., Gratton J.-P., Lin M., Li K.-X., Michell B., Kemp B. E., Rodman D., Sessa W. C. (2002) Localization of endothelial nitric-oxide synthase phosphorylated on serine 1179 and nitric oxide in Golgi and plasma membrane defines the existence of two pools of active enzyme. J. Biol. Chem. 277, 4277–4284 11729179 10.1074/jbc.M106302200

[30] Schmitz M. H. A., Held M., Janssens V., Hutchins J. R. A., Hudecz O., Ivanova E., Goris J., Trinkle-Mulcahy L., Lamond A. I., Poser I., Hyman A. A., Mechtler K., Peters J.-M., Gerlich D. W. (2010) Live-cell imaging RNAi screen identifies PP2A-B55alpha and importin-beta1 as key mitotic exit regulators in human cells. Nat. Cell Biol. 12, 886–893 20711181 10.1038/ncb2092 PMC3839080

[31] Grallert A., Boke E., Hagting A., Hodgson B., Connolly Y., Griffiths J. R., Smith D. L., Pines J., Hagan I. M. (2015) A PP1-PP2A phosphatase relay controls mitotic progression. Nature 517, 94–98 25487150 10.1038/nature14019 PMC4338534

[32] Olszewska-Pazdrak B., Hein T. W., Olszewska P., Carney D. H. (2009) Chronic hypoxia attenuates VEGF signaling and angiogenic responses by downregulation of KDR in human endothelial cells. Am. J. Physiol. Cell Physiol. 296, C1162–C1170 19244479 10.1152/ajpcell.00533.2008

[33] Toporsian M., Govindaraju K., Nagi M., Eidelman D., Thibault G., Ward M. E. (2000) Downregulation of endothelial nitric oxide synthase in rat aorta after prolonged hypoxia in vivo. Circ. Res. 86, 671–675 10747003 10.1161/01.res.86.6.671

[34] Murata T., Sato K., Hori M., Ozaki H., Karaki H. (2002) Decreased endothelial nitric-oxide synthase (eNOS) activity resulting from abnormal interaction between eNOS and its regulatory proteins in hypoxia-induced pulmonary hypertension. J. Biol. Chem. 277, 44085–44092 12185080 10.1074/jbc.M205934200

[35] McQuillan L. P., Leung G. K., Marsden P. A., Kostyk S. K., Kourembanas S. (1994) Hypoxia inhibits expression of eNOS via transcriptional and posttranscriptional mechanisms. Am. J. Physiol. 267, H1921–H1927 7526714 10.1152/ajpheart.1994.267.5.H1921

[36] Atkuri K. R., Herzenberg L. A., Herzenberg L. A. (2005) Culturing at atmospheric oxygen levels impacts lymphocyte function. Proc. Natl. Acad. Sci. USA 102, 3756–3759 15738407 10.1073/pnas.0409910102 PMC553335

[37] Tiede L. M., Cook E. A., Morsey B., Fox H. S. (2011) Oxygen matters: tissue culture oxygen levels affect mitochondrial function and structure as well as responses to HIV viroproteins. Cell Death Dis. 2, e246 22190005 10.1038/cddis.2011.128 PMC3253381

[38] Stacpoole S. R. L., Bilican B., Webber D. J., Luzhynskaya A., He X. L., Compston A., Karadottir R., Franklin R. J. M., Chandran S. (2011) Derivation of neural precursor cells from human ES cells at 3% O(2) is efficient, enhances survival and presents no barrier to regional specification and functional differentiation. Cell Death Differ. 18, 1016–1023 21274009 10.1038/cdd.2010.171 PMC3091847

[39] Li T.-S., Cheng K., Malliaras K., Matsushita N., Sun B., Marbán L., Zhang Y., Marbán E. (2011) Expansion of human cardiac stem cells in physiological oxygen improves cell production efficiency and potency for myocardial repair. Cardiovasc. Res. 89, 157–165 20675298 10.1093/cvr/cvq251 PMC3002866

[40] Stacpoole S. R. L., Webber D. J., Bilican B., Compston A., Chandran S., Franklin R. J. M. (2013) Neural precursor cells cultured at physiologically relevant oxygen tensions have a survival advantage following transplantation. Stem Cells Transl. Med. 2, 464–472 23677643 10.5966/sctm.2012-0144 PMC3673758

[41] El Alami M., Viña-Almunia J., Gambini J., Mas-Bargues C., Siow R. C. M., Peñarrocha M., Mann G. E., Borrás C., Viña J. (2014) Activation of p38, p21, and NRF-2 mediates decreased proliferation of human dental pulp stem cells cultured under 21% O2. Stem Cell Rep. 3, 566–573 10.1016/j.stemcr.2014.08.002 PMC4223702 25358785

[42] Yamamoto K., Korenaga R., Kamiya A., Qi Z., Sokabe M., Ando J. (2000) P2X(4) receptors mediate ATP-induced calcium influx in human vascular endothelial cells. Am. J. Physiol. Heart Circ. Physiol. 279, H285–H292 10899068 10.1152/ajpheart.2000.279.1.H285

[43] Ando J., Komatsuda T., Kamiya A. (1988) Cytoplasmic calcium response to fluid shear stress in cultured vascular endothelial cells. In Vitro Cell. Dev. Biol. 24, 871–877 3170444 10.1007/BF02623896

[44] Ayajiki K., Kindermann M., Hecker M., Fleming I., Busse R. (1996) Intracellular pH and tyrosine phosphorylation but not calcium determine shear stress-induced nitric oxide production in native endothelial cells. Circ. Res. 78, 750–758 8620594 10.1161/01.res.78.5.750

[45] Fleming I., Bauersachs J., Fisslthaler B., Busse R. (1998) Ca2+-independent activation of the endothelial nitric oxide synthase in response to tyrosine phosphatase inhibitors and fluid shear stress. Circ. Res. 82, 686–695 9546377 10.1161/01.res.82.6.686

[46] Lantoine F., Iouzalen L., Devynck M. A., Millanvoye-Van Brussel E., David-Dufilho M. (1998) Nitric oxide production in human endothelial cells stimulated by histamine requires Ca2+ influx. Biochem. J. 330, 695–699 9480877 10.1042/bj3300695 PMC1219192

[47] Østergaard L., Simonsen U., Eskildsen-Helmond Y., Vorum H., Uldbjerg N., Honoré B., Mulvany M. J. (2009) Proteomics reveals lowering oxygen alters cytoskeletal and endoplasmatic stress proteins in human endothelial cells. Proteomics 9, 4457–4467 19670369 10.1002/pmic.200800130

[48] Persechini A., Stemmer P. M. (2002) Calmodulin is a limiting factor in the cell. Trends Cardiovasc. Med. 12, 32–37 11796242 10.1016/s1050-1738(01)00144-x

[49] Busse R., Mülsch A. (1990) Calcium-dependent nitric oxide synthesis in endothelial cytosol is mediated by calmodulin. FEBS Lett. 265, 133–136 1694782 10.1016/0014-5793(90)80902-u

[50] Woods A., Dickerson K., Heath R., Hong S.-P., Momcilovic M., Johnstone S. R., Carlson M., Carling D. (2005) Ca2+/calmodulin-dependent protein kinase kinase-beta acts upstream of AMP-activated protein kinase in mammalian cells. Cell Metab. 2, 21–33 16054096 10.1016/j.cmet.2005.06.005

[51] Moreno C. S., Park S., Nelson K., Ashby D., Hubalek F., Lane W. S., Pallas D. C. (2000) WD40 repeat proteins striatin and S/G(2) nuclear autoantigen are members of a novel family of calmodulin-binding proteins that associate with protein phosphatase 2A. J. Biol. Chem. 275, 5257–5263 10681496 10.1074/jbc.275.8.5257 PMC3505218

[52] Janssens V., Jordens J., Stevens I., Van Hoof C., Martens E., De Smedt H., Engelborghs Y., Waelkens E., Goris J. (2003) Identification and functional analysis of two Ca2+-binding EF-hand motifs in the B"/PR72 subunit of protein phosphatase 2A. J. Biol. Chem. 278, 10697–10706 12524438 10.1074/jbc.M211717200

[53] Davis A. J., Yan Z., Martinez B., Mumby M. C. (2008) Protein phosphatase 2A is targeted to cell division control protein 6 by a calcium-binding regulatory subunit. J. Biol. Chem. 283, 16104–16114 18397887 10.1074/jbc.M710313200 PMC2414307

[54] Lu Q., Pallas D. C., Surks H. K., Baur W. E., Mendelsohn M. E., Karas R. H. (2004) Striatin assembles a membrane signaling complex necessary for rapid, nongenomic activation of endothelial NO synthase by estrogen receptor alpha. Proc. Natl. Acad. Sci. USA 101, 17126–17131 15569929 10.1073/pnas.0407492101 PMC534607

[55] Baharians Z., Schönthal A. H. (1998) Autoregulation of protein phosphatase type 2A expression. J. Biol. Chem. 273, 19019–19024 9668082 10.1074/jbc.273.30.19019

[56] Sanders S. P., Zweier J. L., Kuppusamy P., Harrison S. J., Bassett D. J., Gabrielson E. W., Sylvester J. T. (1993) Hyperoxic sheep pulmonary microvascular endothelial cells generate free radicals via mitochondrial electron transport. J. Clin. Invest. 91, 46–52 8380815 10.1172/JCI116198 PMC329993

[57] Chandel N. S., Maltepe E., Goldwasser E., Mathieu C. E., Simon M. C., Schumacker P. T. (1998) Mitochondrial reactive oxygen species trigger hypoxia-induced transcription. Proc. Natl. Acad. Sci. USA 95, 11715–11720 9751731 10.1073/pnas.95.20.11715 PMC21706

[58] Palacios-Callender M., Hollis V., Mitchison M., Frakich N., Unitt D., Moncada S. (2007) Cytochrome c oxidase regulates endogenous nitric oxide availability in respiring cells: a possible explanation for hypoxic vasodilation. Proc. Natl. Acad. Sci. USA 104, 18508–18513 18003892 10.1073/pnas.0709440104 PMC2141807

[59] Beckman J. S., Beckman T. W., Chen J., Marshall P. A., Freeman B. A. (1990) Apparent hydroxyl radical production by peroxynitrite: implications for endothelial injury from nitric oxide and superoxide. Proc. Natl. Acad. Sci. USA 87, 1620–1624 2154753 10.1073/pnas.87.4.1620 PMC53527

