# Supplementary material for: A PP2A-mediated feedback mechanism controls Ca2+-dependent NO synthesis under physiological oxygen
Source: FASEB J. 2017 Jul 31;31(12):5172–83. doi: 10.1096/fj.201700211R (PMC5690389; doi:10.1096/fj.201700211R)
Supplement: Supplemental Data [file supp_31_12_5172__index.html]

A PP2A-mediated feedback mechanism controls Ca2+-dependent NO synthesis under physiological oxygen — A PP2A-mediated feedback mechanism controls Ca2+-dependent NO synthesis under physiological oxygen — Supplemental Data 

# A PP2A-mediated feedback mechanism controls Ca2+-dependent NO synthesis under physiological oxygen

## Supplemental Data

- Supplemental Data
