# Supplementary material for: A PP2A-mediated feedback mechanism controls Ca2+-dependent NO synthesis under physiological oxygen
Source: FASEB J. 2017 Jul 31;31(12):5172–83. doi: 10.1096/fj.201700211R (PMC5690389; doi:10.1096/fj.201700211R)
Supplement: Supplemental Data [file supp_fj.201700211R_Supplemental_Figures.pdf]

Supplemental Data Unit 1

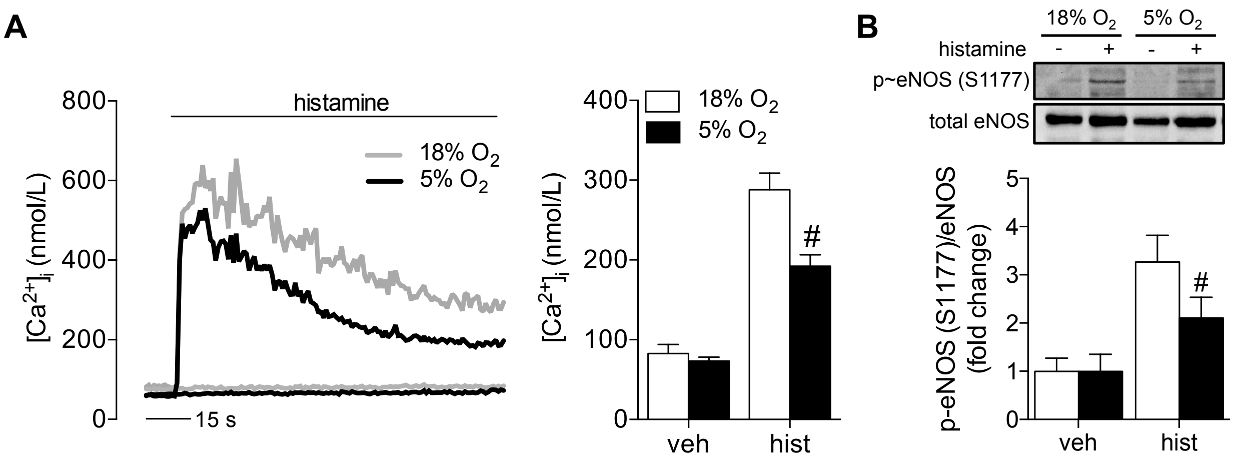

Figure S1. Effects of physiological O_2 levels on Ca^{2+} mobilization and eNOS phosphorylation in human coronary artery endothelial cells.

HCAEC were cultured under standard (18%) or physiological (5%) O_2 levels for at least 5 days. (**A**) Basal and histamine stimulated intracellular Ca^{2+} levels, shown as representative traces and the mean plateau values (115-120 s). (**B**) Histamine stimulated (10 µmol/L, 2 min) eNOS-S1177 phosphorylation expressed relative to total eNOS and loading control β-actin (not shown). Data denote mean ± S.E.M., n=4-5 different cultures, **P<0.01, ***P<0.001 vs vehicle, #P<0.05 vs 18% O_2. Treatment with histamine was significant vs control (P<0.05) under both 18% and 5% O_2.

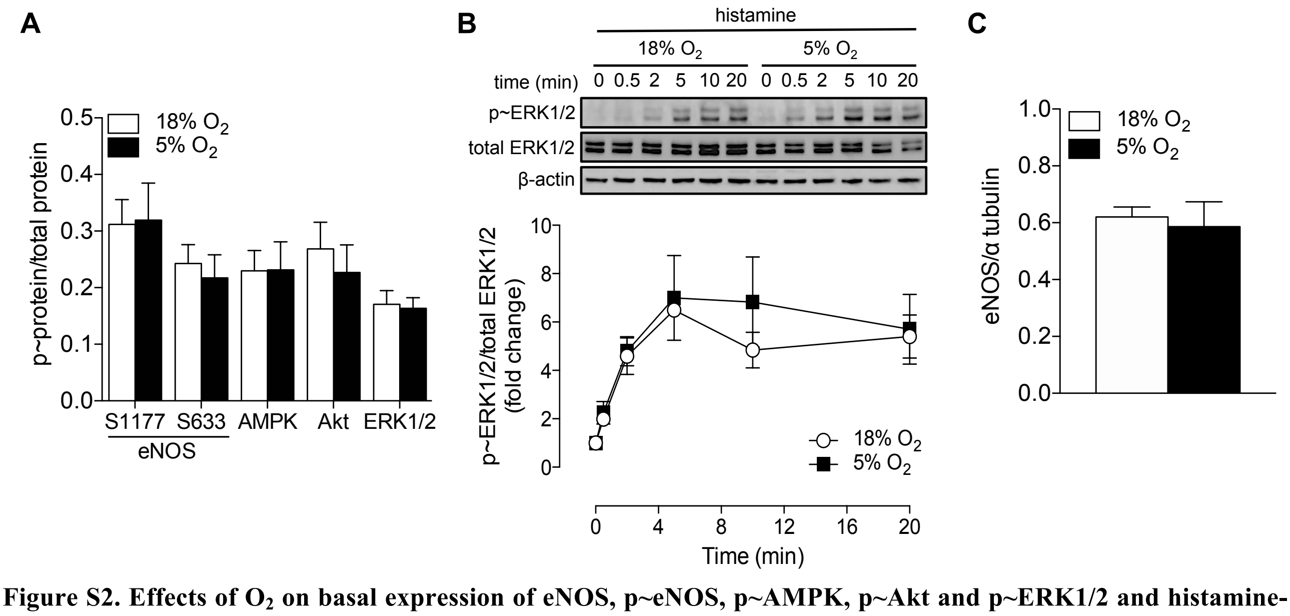

Figure S2. Effects of O_2 on basal expression of eNOS, p~eNOS, p~AMPK, p~Akt and p~ERK1/2 and histamine-stimulated ERK1/2 phosphorylation in HUVEC.

Cells were cultured under standard (18%) or physiological (5%) O_2 levels for at least 5 days. (**A**) Basal p~eNOS (Ser1177 and Ser633), p~AMPK p~Akt and p~ERK1/2 phosphorylation relative to respective total kinase protein levels and loading control β-actin (not shown). (**B**) HUVEC were stimulated with histamine (10 µmol/L) for 0-20 min and cell lysates immunoblotted for p~ERK1/2 relative to total ERK1/2 and loading control β-actin. **C**, eNOS expression in cells cultured under 18% or 5% O_2. Data denote mean ± S.E.M., n=5 different donors.

Supplemental Data Unit 2

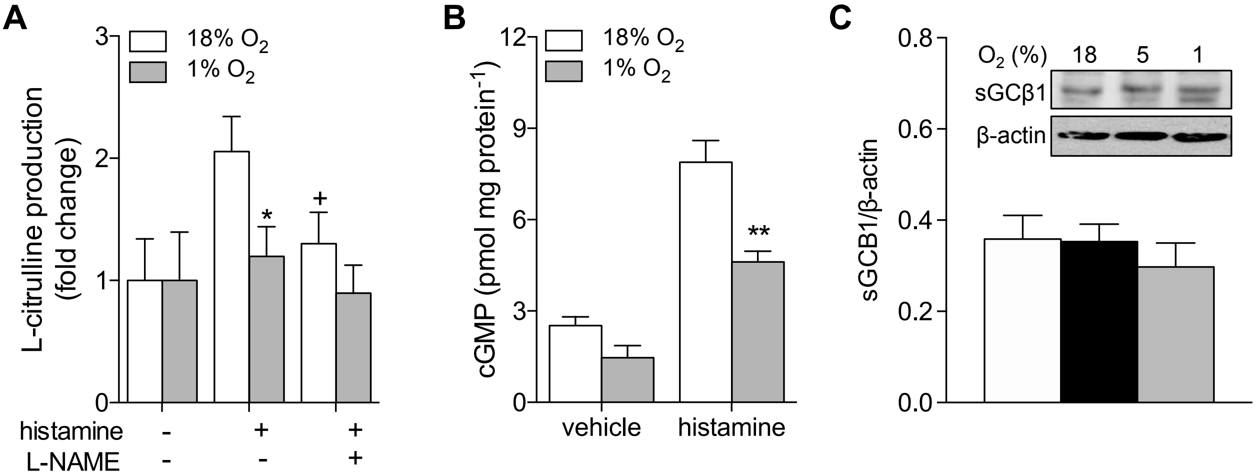

Figure S3. eNOS activity and generation of cGMP in HUVEC under 1% O_2.
HUVEC were cultured under standard (18%) or hypoxic (1%) O_2 levels for at least 5 days. (**A**) Histamine (10 µmol/L, 5 min) stimulated conversion of L-[^{3}H]arginine to L-[^{3}H]citrulline (L-NAME-inhibitable, 100 µmol/L) as a measurement of eNOS activity, (**B**) Histamine stimulated cGMP generation, and **C**, soluble guanylate cyclase β1 subunit expression in cells cultured under 18, 5 and 1% O_2. Data represent mean ± S.E.M. from 3-6 different donors. *P<0.05, **P<0.01 vs 18% O_2, +P<0.05 vs histamine alone. Treatment with histamine was significant (P<0.05) vs respective control in both panels A and B at 18% O_2, and only in panel B at 1% O_2.

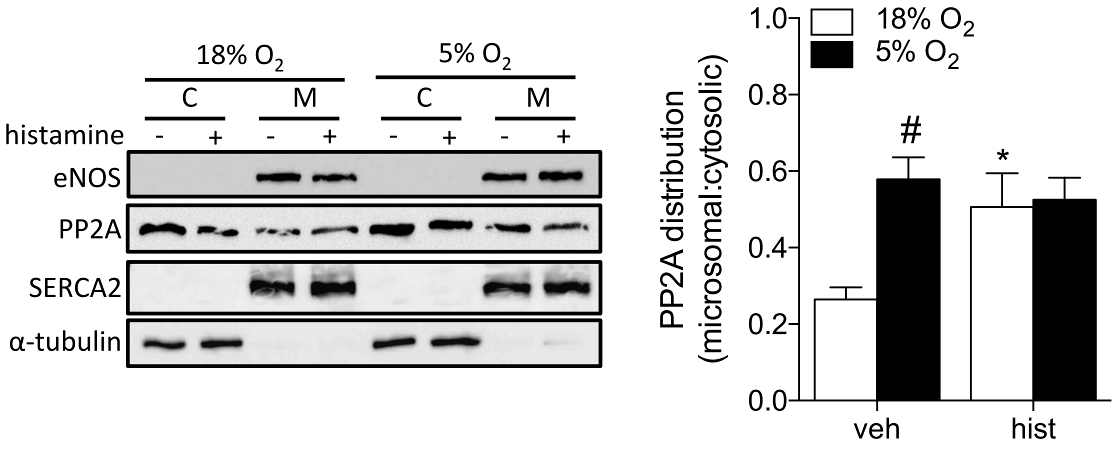

Figure S4. PP2A expression in cytosolic and microsomal fractions following histamine stimulation under physiological O_2 levels.
HUVEC were cultured under standard (18%) or physiological (5%) O_2 levels for at least 5 days and then stimulated with histamine (10 µmol/L, 5 min). (**A**) Lysates were separated into cytosolic (C) and microsomal (M) fractions by ultracentrifugation and presence of PP2A-A and eNOS assessed by immunoblot analysis (**B**). Purity of the microsomal fraction was confirmed by absence of α-tubulin and presence of both SERCA2 and caveolin-1 (data not shown). (**B**) Quantification of PP2A microsomal distribution relative to cytosolic. Data denote the mean ± S.E.M. from 5 different donors. *P<0.05 vs veh, #P<0.05 vs 18% O_2.

Supplemental Data Unit 3

A

Variable	Definition	Value	Reference
K_1	Donor decomposition rate constant (s^{-1})	1.92×10^{-5}	(Keefer 1996)
K_2	NO autoxidation rate constant (s^{-1})	*Aqueous* 6.6×10^{-6}<br>*Lipid* 8.8×10^{-7}	(Liu 1998)
E_{NO}	Stoichiometry of NO release	2	(Keefer 1996)
C_D	Donor concentration (M)	1×10^{-6} - 5×10^{-4}	
O_2	Oxygen concentration (M)	3.37×10^{-4} - 1.35×10^{-6}	
t	Time (s)	0 - 600	

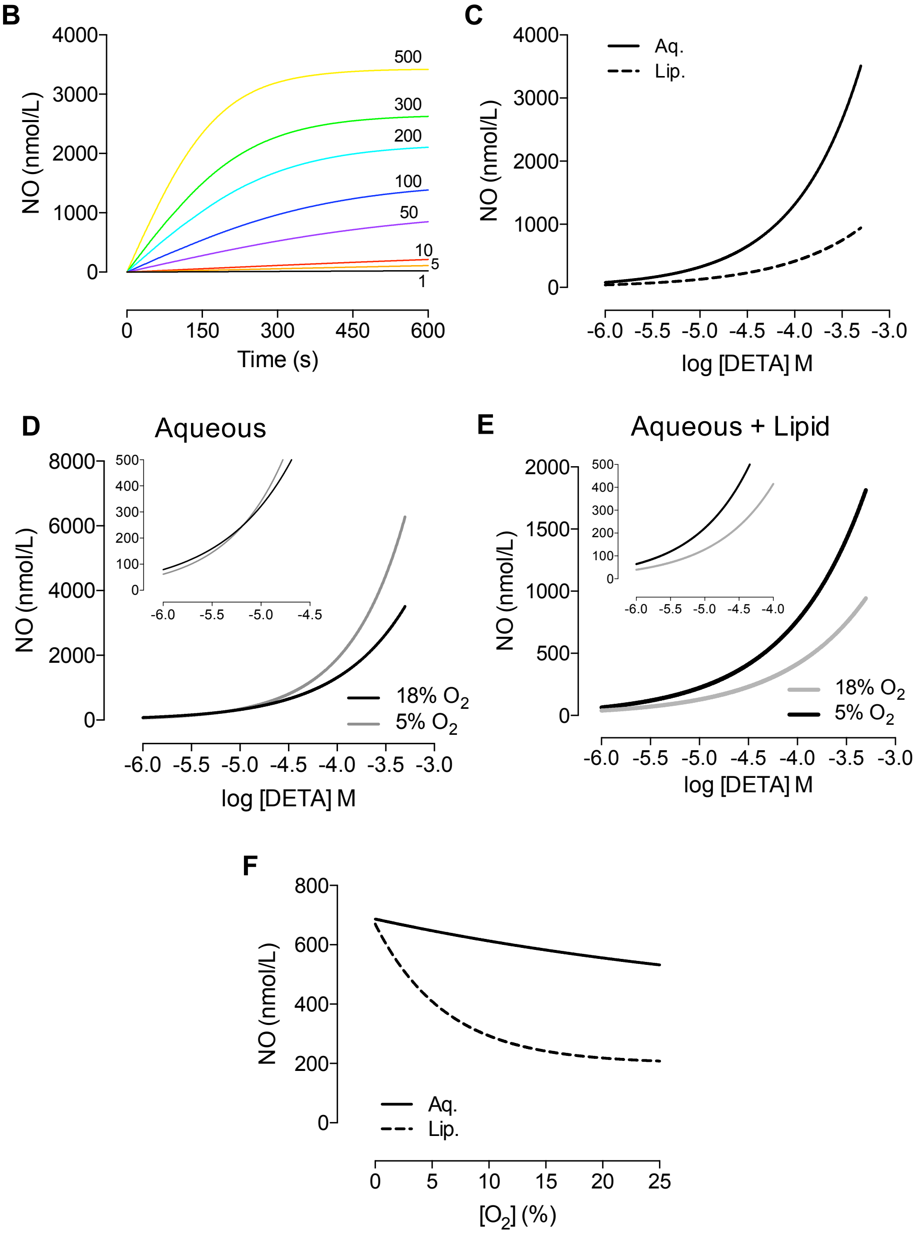

Figure S5. Modeling of NO concentrations generated from DETA NONOate decomposition.
(**A**) Parameters used to simulate NO concentrations and description of each parameter and numerical values used. (**B**) Relationship between NO and DETA NONOate (DETA) concentrations over a 10 min period. (**C**) The effect of the presence of lipids (40 mg/ml lipid micelles)[21] on NO concentration. The effect of 5% O_2 on DETA derived NO concentrations in an aqueous environment (**D**) or in the presence of lipids (**E**). Inset highlights results under a physiological range of NO output within the healthy vasculature. (**F**) Effect of varying O_2 levels on DETA-derived NO concentrations calculated using variables for aqueous and lipid environments.
